# Evaluation of antibodies in cerebrospinal fluid for the diagnosis of tick-borne encephalitis in dogs

**DOI:** 10.1186/s13028-021-00597-9

**Published:** 2021-08-26

**Authors:** Yvonne Alnefelt, Sofie Van Meervenne, Katarina Varjonen, Anna Tidholm, Cecilia Rohdin

**Affiliations:** 1 Anicura Albano Small Animal Hospital, 182 36 Danderyd, Sweden; 2 Anicura Kalmar Small Animal Hospital, 392 45 Kalmar, Sweden; 3 Department of Clinical Sciences, Swedish University of Agricultural Sciences, 75007 Uppsala, Sweden

**Keywords:** Canine, Meningoencephalitis, Meningoencephalomyelitis, Serology, Virus

## Abstract

Tick-borne encephalitis (TBE) is caused by the neurotropic tick-borne encephalitis virus (TBEV). In dogs, this virus may affect the central nervous system (CNS), causing meningoencephalitis, meningomyelitis, radiculitis or any combination of these. Diagnosis of TBE relies on a combination of clinical signs of CNS disease and laboratory findings, including CSF pleocytosis and serum TBEV antibody titers. Exposure to TBEV does not necessarily cause clinical disease, and seroprevalence has been reported as high as 40% in endemic areas. This causes concerns of over-diagnosing TBE in dogs with CNS disease. By examining TBEV antibodies in dogs with and without neurological disease in a TBEV endemic area, this study aimed to evaluate the diagnostic value of TBEV antibodies in the cerebrospinal fluid (CSF) in dogs. Eighty-nine dogs were included in the study, 56 with neurological disease and 33 neurologically normal control dogs. A positive TBEV CSF and serum IgG antibody titer (> 126 U/mL) was found in 3/89 dogs (3.4%). A positive serum TBEV antibody titer was found in 11 of the 89 dogs (12.4%). None of the control dogs showed a positive CSF antibody titer, whilst two showed positive serum concentrations. A positive CSF IgG antibody titer supports a clinical diagnosis of TBE in patients with acute onset of CNS disease and may help reduce the risk of over-diagnosis.

## Findings

Tick-borne encephalitis (TBE) is caused by the neurotropic tick-borne encephalitis virus (TBEV) [1, 2]. In Europe, this virus is transmitted mainly by *Ixodes ricinus* ticks [3]. TBEV affects the central nervous system (CNS), most commonly the brain but may also involve the spinal cord and nerve roots, causing meningoencephalitis, meningomyelitis or radiculitis [4]. Canine TBE has been characterized by clinical signs that are almost similar to TBE in humans, but with lower morbidity and a higher mortality rate compared to humans [3, 5–7]. Although the prognosis for canine TBE has been described as poor, affected dogs may recover without complications [8]. A diagnosis of TBE relies on a combination of clinical and laboratory findings [9]. In contrast to other viral infections, polymerase chain reaction (PCR) methods are rarely useful for the in vivo diagnosis of TBE since by the time neurological symptoms become manifest, the virus has already been cleared from the blood and the cerebrospinal fluid (CSF). In humans, laboratory confirmation of TBE is based on CSF analysis and evaluation of TBEV specific antibody titers in serum and/or CSF [10, 11]. TBEV antibody testing of CSF is considered a reliable diagnostic tool, and TBEV specific antibodies are found in the majority of human patients [9, 12, 13]. In veterinary medicine, the clinical diagnosis is commonly based on IgG seropositivity and CSF pleocytosis in dogs with signs of acute CNS disease localized to the brain [3]. However, as seroconversion is common in dogs, with reported seroprevalences of TBEV up to 40% in the Nordic countries [14–16], seropositivity becomes of questionable value [3, 17]. Analysis of TBEV antibody titers in CSF has therefore been suggested as an acute diagnostic test for dogs with presumed TBE [17, 18]. By examining TBEV antibodies in CSF in a group of dogs, with and without neurological signs in a TBEV endemic area, this study aimed to evaluate the diagnostic value of antibodies in CSF. We hypothesized that if present, TBEV antibody titers are positive in CSF from dogs presenting with an acute onset of signs localized to the brain.

Privately owned dogs were prospectively recruited between 2012 and 2017 at Anicura Albano Animal Hospital, Stockholm, Sweden. Ethical approval from Animal Ethics Committee of Sweden was obtained, and dogs were only included if owners had given consent to participate. Dogs with neurological disease were recruited from patients presenting to the neurology service, and dogs without neurological disease, were recruited from dogs presenting for euthanasia due to non-neurological disease, through the emergency service at Anicura Albano Animal Hospital. All dogs underwent a clinical examination and dogs with CNS disease also underwent a neurological examination by a board-certified neurologist or a veterinarian in training to become a Swedish specialist in neurology in dogs and cats. Dogs with neurological disease were divided into two groups. Group A included dogs admitted with an acute onset of neurological signs localised to the brain and group B included dogs with signs of other neurological localization or chronic (> 2 weeks) neurological signs localised to the brain. Group C included dogs euthanized for reasons unrelated to a neurological disorder and without neurological signs. For dogs with neurological disease (group A and B), CSF and blood sampling were performed as part of their routine clinical work-up. In group A (n  =  20) and B (n  =  36) all dogs had CSF cell count and protein concentration analyzed. Polymerase chain reaction was used to analyze for infectious diseases (Canine distemper virus, *Neospora caninum*, *Toxoplasma gondii* and *Anaplasma phagocytophilium*) for 10 dogs in Group A and 20 dogs in Group B. For dogs in group C, admitted for euthanasia, blood sampling was performed as the venous catheter was inserted and the CSF was taken immediately post-mortem. The CSF was taken from the cerebellomedullary cistern in all dogs and samples were analyzed within 30 min of collection. Cerebrospinal fluid was interpreted to have pleocytosis if there were  >  5 nucleated cells/µL, and pleocytosis was classified by the primary cell population if one cell type comprised  >  50%. Protein concentration was considered to be increased if  >  0.25 g/L at the cerebello-medullary cistern [19]. Serum and CSF samples from all dogs were immediately frozen and stored at − 18 °C. Samples were then packed on dry ice and shipped by express delivery to the veterinary diagnostic laboratory Laboklin (Bad Kissingen, Germany) for analysis for IgG antibodies against TBEV (IgG TBEV). The method used was the commercially available enzyme-linked immunosorbent assay (ELISA) Immunozyme FSME (TBE) IgG^a^. An ELISA cut-off value of 126 U/mL for both serum and CSF was used according to the manufacturer’s recommendation at the time the study was initiated.

Descriptive statistics were used for presenting data including signalment, TBEV antibody levels in serum and CSF, CSF pleocytosis and CSF protein concentration. Continuous variables are presented as median and interquartile range (IQR).

A total of 89 dogs were included in the study. Information on signalment and TBEV antibody titers in CSF and serum of included dogs is summarised in Table 1. A positive TBEV CSF antibody titer was found in three of the 89 dogs (3.4%). These three dogs belonged to group A and the individual TBEV CSF antibody titer of these dogs was 131.8, 194.3 and 188.0 U/mL respectively. The three dogs, a Rottweiler, a Labrador and a Bedlingtonterrier, lived in the Stockholm archipelago, a highly TBE endemic area, and all presented with fever, decreased mentation, hyperesthesia, ataxia, hypermetria, disorientation and seizures. Two (2/3) dogs presented with CSF pleocytosis, the third dog with an unremarkable CSF was on steroids before sampling. All three dogs also presented with a positive TBEV serum antibody titer (142.8, 415.8 and 169.5 U/mL respectively).

**Table 1:** Information on signalment, TBEV antibody titers in CSF and serum, and results of CSF analyzis of all dogs (n  =  89) included in the study

Variable	Group A	Group B	Group C
Total number of dogs 89	20	36	33
Sex			
Female	12 (60.0%)	15 (41.7%)	8 (24.2%)
Neutered female	2 (10.0%)	2 (5.6%)	4 (12.1%)
Male	3 (15.0%)	17 (47.2%)	14 (42.4%)
Neutered male	3 (15.0%)	2 (5.6%)	7 (21.2%)
Median age (years)	4.9 IQR 2.0–5.5	2.0 IQR 1.0–7.3	10.0 IQR 7.6–12.4
Median body weight (kg)	17.0 IQR 6.0–27.0	11.0 IQR 7.6–21.4	26.0 IQR 9.6–30.8
Median TBEV ab (U/mL) CSF	26.8 IQR 20.2–34.8	18.8 IQR 15.3–27.1	22.4 IQR 16.1–33.4
Range TBEV ab (U/mL) CSF	2.7–194.3	13.6–115.6	9.6–81.3
Number of dogs with CSF ab > 126 U/mL	3 (15.0%)	0	0
Median TBEV ab (U/mL) serum	26.3 IQR 20.1–129.3	22.3 IQR 16.2–38.5	40.9 IQR 24.6–76.5
Range TBEV ab (U/mL) serum	20.1–930.3	16.2–430.7	15.1–171.1
Number of dogs with serum ab > 126 U/mL	5 (25.0%)	4 (11.1%)	2 (6.1%)
Number of dogs with CSF pleocytosis	13 (65.0%)	11 (30.6%)	Not analysed
Median number of leukocytes in CSF	16.0 IQR 2.5–40.5	2.5 IQR 0.0–7.5	Not analysed
Median protein (g/L) in CSF	0.32 IQR 0.18–0.86	0.22 IQR 0.13–0.43	Not analysed

Data are presented as median and IQR

*CSF* cerebrospinal fluid; *IQR* interquartile range; *TBEV* tick-borne encephalitis virus; *ab* antibody

A positive serum TBEV antibody titer was found in 11 of the 89 dogs (12.4%); 5 belonged to group A; 4 to group B; and 2 to group C. The two additional dogs from group A presented with central vestibular signs without fever, the dogs from group B presented with optic neuritis (n  =  1), neoplasia (n  =  1), polyneuropathy (involving cranial nerves) (n  =  1) and steroid responsive meningitis-arteritis (n  =  1). All PCR tests for other infectious diseases in CSF, including the CSF samples from the three dogs with a positive CSF TBEV antibody titer, were negative. The three TBEV CSF antibody positive dogs were all treated with immunosuppressant doses of corticosteroids between two and three weeks and all three dogs survived.

Due to the low number of CSF positive dogs in this study, the accurate diagnostic value of TBEV antibodies in CSF could not be determined. However, a positive TBEV antibody titer in CSF was a rare finding and as hypothesized only included dogs presented with an acute onset of neurological sings localized to the brain. Of the eight dogs from group B and C (n  =  69) that presented with a positive serum TBEV antibody titer none showed a positive CSF TBEV antibody titer. In addition, two dogs without neurological signs presented with a positive serum TBEV antibody titer. The diagnosis of TBE should be based on clinical and laboratory findings with CSF pleocytosis confirming CNS involvement and serological testing proving exposure to the TBEV. The Immunozyme FSM IgG ELISA has been shown to be a highly specific test for the detection of antibodies in serum against TBEV in dogs [20] however its sensitivity and specificity for TBEV antibodies in CSF has not been determined. To the authors’ knowledge, this is the first study measuring TBEV antibody titers in serum and CSF of both neurologically affected and unaffected dogs in a TBE endemic area. We used the manufacturers recommended antibody cut-off value for TBEV positivity of 126 U/mL in CSF which is in agreement with a previous study of TBE in dogs [17]. The manufacturer has since changed the cut-off value to  >  20 U/mL. With a cut-off value of TBEV antibodies of  >  20 U/mL in the CSF, the majority of dogs included in this study (57.3%), regardless of neurological status, would have been considered TBEV positive. However, TBEV CSF antibody testing would then be of no clinical value, and erroneous clinical diagnoses might be made depending on the cut off value provided by the laboratory.

It was a limitation to our study that the CSF TBEV positive dogs were not confirmed by neutralization test. We cannot rule out the possibility that the antibodies passively diffused from serum into CSF due to a blood–brain-barrier disruption. However, the study included dogs presenting with an acute meningoencephalitis and even higher serum TBEV antibody titers with low CSF TBEV antibodies. Also, as all three dogs survived, histopathological examination including RT-qPCR was not an option, and paired serum samples were not analysed as the dogs were not available for follow up sampling.

## Conclusions

The present study investigated CSF IgG antibody titers in dogs with and without neurological signs and found that only in dogs with acute neurological signs from the brain, TBEV antibody titers were positive in both serum and CSF. If only performing serum TBEV antibody titers in dogs under suspicion for TBE, the risk of over diagnosing TBE is imminent.

## Data Availability

The datasets used and/or analysed during the current study are available from the corresponding author on reasonable request.

## Abbreviations

CNS: Central nervous system
CSF: Cerebrospinal fluid
ELISA: Enzyme-linked immunosorbent assay
IgG: Immunoglobulin G
IgM: Immunoglobulin M
PCR: Polymerase chain reaction
TBE: Tick-borne encephalitis
TBEV: Tick-borne encephalitis virus
